# Effects of Small-Sided Game Interventions on the Technical Execution and Tactical Behaviors of Young and Youth Team Sports Players: A Systematic Review and Meta-Analysis

**DOI:** 10.3389/fpsyg.2021.667041

**Published:** 2021-05-07

**Authors:** Filipe Manuel Clemente, Rodrigo Ramirez-Campillo, Hugo Sarmento, Gibson Moreira Praça, José Afonso, Ana Filipa Silva, Thomas Rosemann, Beat Knechtle

**Affiliations:** ^1^Escola Superior Desporto e Lazer, Instituto Politécnico de Viana Do Castelo, Rua Escola Industrial e Comercial de Nun'Álvares, Viana Do Castelo, Portugal; ^2^Instituto de Telecomunicações, Delegação da Covilhã, Lisboa, Portugal; ^3^Department of Physical Activity Sciences, Universidad de Los Lagos, Santiago, Chile; ^4^Centro de Investigación en Fisiología del Ejercicio, Facultad de Ciencias, Universidad Mayor, Santiago, Chile; ^5^Research Unit for Sport and Physical Activity (CIDAF), Faculty of Sport Sciences and Physical Education, University of Coimbra, Coimbra, Portugal; ^6^Sports Department, Universidade Federal de Minas Gerais, Belo Horizonte, Brazil; ^7^Centre for Research, Education, Innovation and Intervention in Sport, Faculty of Sport of the University of Porto, Porto, Portugal; ^8^N2i, Polytechnic Institute of Maia, Maia, Portugal; ^9^The Research Centre in Sports Sciences, Health Sciences and Human Development (CIDESD), Vila Real, Portugal; ^10^Institute of Primary Care, University of Zurich, Zurich, Switzerland; ^11^Medbase St. Gallen Am Vadianplatz, St. Gallen, Switzerland

**Keywords:** football, Soccer, athletic performance, youth sports, decision-making, motor skills

## Abstract

**Background:** Small-sided games (SSGs) are an adjusted form of official games that are often used in training scenarios to introduce a specific tactical issue to team sports players. Besides the acute effects of SSGs on players' performance, it is expectable that the consistent use of these drill-based games induces adaptations in the technical execution and tactical behaviors of youth team sports players.

**Objective:** This systematic review with meta-analysis was conducted to assess the effects of SSG programs on the technical execution and tactical behaviors of young and youth team sports players.

**Data Sources:** The data sources utilized were PubMed, Scopus, SPORTDiscus, and Web of Science.

**Study Eligibility Criteria:** (i) Young and youth team sports players (i.e., < 18 years old) of any sex or skill level, without injury, illness, or other clinical conditions; (ii) SSGs-based programs not restricted to the duration and a minimum of one weekly training session; (iii) passive or active control groups; (iv) pre-post interventions values of technical execution and/or tactical behavior; (v) randomized and non-randomized controlled trials; and (vi) peer-reviewed original full-text studies written in English, Portuguese and/or Spanish.

**Results:** The database search initially yielded 803 titles. From those, six articles were eligible for the systematic review and meta-analysis. None of the included studies presented tactical behavior outcomes. The results showed a small effect of SSGs on technical execution (ES = 0.59; 95% CI = 0.29 to 0.89; *p* < 0.001; *I*^2^ = 0.0%; Egger's test *p* = 0.590) when compared to controls. Sub-group analysis of the training factor revealed similar (*p* = 0.433) moderate (ES = 0.68, four study groups) and small (ES = 0.44, three study groups) improvements in technical execution after >17 and <17 SSG training sessions, respectively.

**Conclusions:** This systematic review and meta-analysis revealed a significant beneficial effect of using SSG training programs for enhancing technical execution in young and youth players. The benefits were similar despite the various numbers of training sessions applied. Further studies should add tactical behaviors as one of the outcomes for controlling the effects of SSG training programs.

## Introduction

Small-sided games (SSGs), are adjusted formats of play, in which the coach modifies the number of players involved, the pitch configuration, or specific rules of the game to introduce a specific tactical issue to team sports players (Davids et al., 2013). The manipulation of these constraints (or conditions) promotes an immediate effect on players' responses, namely in terms of tactical behaviors (Clemente et al., 2020), technical execution (Clemente and Sarmento, 2020), or physiological and physical demands (Clemente, 2016; Sarmento et al., 2018). The consistent use of these drills over several weeks will likely promote changes in players' tactical or technical skills (Práxedes et al., 2018a) or their fitness status (Hammami et al., 2018; Moran et al., 2019a; Clemente et al., 2021).

SSGs can play an important role in young and youth team sports training, as they develop multidimensional factors (e.g., technical/tactical and physical/physiological factors) (Fernández-Espínola et al., 2020). Indeed, these drills may apply a multidimensional stimulus to players (e.g., technical/tactical, physiological/physical) while keeping them attentive to specific tactical aspects of the game (Ometto et al., 2018). The effects of SSGs on young and youth team sports players vary based on different factors (e.g., age-group, expertise level, fitness status) (Olthof et al., 2018; Práxedes et al., 2018b). However, SSGs may promote changes in players' tactical behaviors and technical execution since they are playing a modified version of the game that maintains the fundamental dynamics and specificities of their sport (Davids et al., 2013).

Young and youth players are sensitive to learning and developing game-related skills (Serra-Olivares et al., 2016), thus promoting qualitative acquisition leading to expertise (Silva et al., 2020). In the particular case of team sports, skill-related outcomes are associated with players' technical abilities (or execution), tactical behaviors, and the decisions related to tactical behaviors (González-Víllora et al., 2015). Usually, technical skill or execution is analyzed using observational instruments that focus on the accuracy of skill-related actions. Meanwhile, tactical behaviors can be coded based on the accomplishment of tactical principles or expectations of attacking and/or defensive behaviors (González-Víllora et al., 2015). Pre-post analyses of such outcomes are often used to monitor the development of young and youth players (Turner et al., 2011).

While maintaining certain dynamics of official games, SSGs also ensure considerable levels of heterogeneity on the players' stimulus (Clemente, 2019). They also provide great intra-player variability across the same format of play (Clemente et al., 2018; Clemente et al., 2019). Thus, despite expected medium-to-long term adaptations in the technical execution or tactical behaviors of young and youth team sports players, it is also likely that these adaptations are not similar for all players. In that sense, there is a need for a systematic review and meta-analysis summarizing the evidence about the effects of SSGs on the technical execution and tactical behaviors of young and youth team sports players against control groups.

Despite the growing number of systematic reviews and meta-analyses published on SSGs (Hammami et al., 2018; Bujalance-Moreno et al., 2019; Clemente and Sarmento, 2020; Clemente et al., 2020), and some dedicated systematic reviews about acute (immediate) effects of SSG in technical and tactical outcomes (Clemente and Sarmento, 2020; Clemente et al., 2020), no meta-analysis has investigated the effects of SSG-based programs on the technical execution and tactical behaviors of young and youth team sports players. A results-derived meta-analysis, along with a related systematic review and analysis of the available literature, may offer coaches an evidence-based overview of the effects of SSGs on young and youth team sport player's technical execution and tactical behaviors. This would also provide practical guidelines regarding potentially effective SSG configurations.

Therefore, the purpose of the present systematic review with meta-analysis was to assess the effects of SSG programs on the technical execution and tactical behaviors of young and youth team sports players. Specifically, within-group and between-group analyses against controls for each outcome are considered in the meta-analysis.

## Methods

This systematic review and meta-analysis followed the Cochrane Collaboration (Green and Higgins, 2005) and PRISMA (Preferred Reporting Items for Systematic Reviews and Meta-analyses) guidelines (Moher et al., 2009). The PICOS approach (Population, Intervention, Comparator, Outcomes, Study design) was followed: (P) youth team sports players (i.e., < 18 years old) from any sex or skill, without injury, illness or other clinical condition; (I) SSGs-based programs no restricted to duration and a minimum of one weekly training sessions; (C) Passive or active control groups; (O) pre-post interventions values of technical execution and/or tactical behavior; and (S) randomized and non-randomized controlled-trials. The protocol was registered with the International Platform of Registered Systematic Review and Meta-Analysis Protocols with the number 202110108 and the DOI number 10.27766/inplasy2021.1.0108.

### Eligibility Criteria

Inclusion and exclusion criteria for this systematic review and meta-analysis can be found in Table 1.

**Table 1:** Inclusion and exclusion criteria.

**Item**	**Inclusion criteria**	**Exclusion criteria**
Population	Youth team sports players (< 18 years old) from any sex or skill, without injury, illness or other clinical condition. Team sports considered, among others: soccer (association football), futsal, handball, volleyball, basketball, hockey, rugby, Australian football, American football, water polo, lacrosse, softball, baseball, korfball.	Team sports players with more than 18 years old. Team sports players in rehabilitation or in return-to-play programs. Other sports than team sports with ball.
Intervention	SSGs-based programs no restricted to duration and a minimum of one weekly training sessions	Other training methods not related to SSGs (e.g., analytical exercises, running exercises). SSGs combined with other training methods will be also included, if any.
Comparator	Passive or active control groups.	Other SSGs training groups.
Outcome	Pre-post intervention values of technical execution (i.e., measures that assess individual ability skill or accuracy of technical execution related with the sport) and/or tactical behavior (i.e., measures that assess individual ability to organize the behavior based on the tactical principles and collective dynamics of the game).	Outcomes not related to technical execution or tactical behavior; no information (e.g., mean; standard deviation) reported for pre- and/or post-intervention (e.g., follow-up excluded).
Study design	Randomized and non-randomized controlled and/or parallel trials, with no significant differences between groups in baseline assessment of the main outcome.	Non-controlled studies or controlled trials in which baseline levels were significantly different between groups for the main outcome.
Additional criteria	Peer reviewed, original, full-text studies written in English, Portuguese and/or Spanish.	Written in other language than those selected. Reviews, letters to editors, trial registrations, proposals for protocols, editorials, book chapters, conference abstracts.

Duplicates were identified using the reference manager software (EndNote^TM^ X9, Clarivate Analytics, Philadelphia, PA, USA). Two authors (FMC and HS) independently performed screening of the title, abstract and reference list of each study to locate potentially relevant studies. Additionally, they reviewed the full version of the papers in detail to identify articles that met the selection criteria and those that were excluded. A discussion was made in the cases of discrepancies regarding the selection process with the participation of a third author (AFS).

### Information Sources

Electronic databases (PubMed, Scopus, SPORTDiscus, and Web of Science) were searched for relevant publications, from inception up to 28th January 2021. Keywords and synonyms were entered in various combinations in all fields: (youth OR young OR “child^*^” OR “adolescent”) AND (“team sport” OR football OR soccer OR futsal OR handball OR volleyball OR basketball OR hockey OR rugby OR cricket OR “water polo” OR lacrosse OR softball OR korfball OR baseball) AND (“small-sided games” OR “sided-games” OR “drill-based games” OR “SSG” OR “conditioned games” OR “small-sided and conditioned games”) AND (“technical” OR “tactic*” OR “skill” OR “ability” OR “behavior*” OR “decision making”). An external expert was contacted to verify the final list of references included in this systematic review and to indicate if there was any study that was not detected through our search.

### Extraction of Data

A data extraction sheet, adapted from the Cochrane Consumers and Communication Review Group's data extraction template (Collaboration, 2016), was used to assess inclusion requirements and subsequently tested on ten randomly selected studies (i.e., pilot testing). This process was conducted by two independent reviewers (FMC and HS). Any disagreement regarding study eligibility was resolved in a discussion between both reviewers and a third author (AFS). Full text articles excluded, with reasons, were recorded. The records were registered in a form created in Microsoft Excel (Microsoft Corporation, Readmon, WA, USA).

### Data Items

Aiming to establish consistency in data analyzing and reporting, only measures that were analyzed three or more times for different articles were included. For technical execution were considered the pre-post intervention outcomes that analyzed the skill level of the player in the specific sport, or the accuracy of skill. For tactical behavior, were considered the pre-post intervention outcomes that assessed individual ability to organize the behavior based on the tactical principles and collective dynamics of the game. The method used for the assessment of technical and tactical outcomes was also extracted. Adverse effects were also extracted as secondary outcome, in case of any reported. Additionally, the following information was extracted from the included studies: (i) number of participants (n), age (years), competitive level (if available) and sex; (ii) the SSGs format and pitch size (if available); (iii) period of intervention (number of weeks) and number of sessions per week (n/w); and (iv) regimen of intervention (work duration, work intensity, modality, relief duration, relief intensity, repetitions and series, between-set recovery).

### Assessment of Methodological Quality

The version 2 of the Cochrane risk-of-bias tool for randomized trials (RoB2) (Sterne et al., 2019) was used to assess the risk of bias in the included randomized-controlled trials. Five dimensions are inspected in this assessment tool: (i) bias arising from the randomization process; (ii) bias due to deviations from intended interventions; (iii) bias due to missing outcome data; (iv) bias in measurement of the outcome; and (v) bias in selection of the reported result. Using RoB2 a qualitative synthesis was performed. Two of the authors (JA and HS) independently assessed the risk of bias. Any disagreement in the rating was resolved through discussion and by a third author (FMC).

The Cochrane risk of bias in non-randomized studies of interventions (ROBINS-I) was used to assess the risk of bias in included non-randomized intervention studies (Sterne et al., 2016). Three domains are analyzed in this assessment tool: (i) pre-intervention (bias due to confounding; bias in selection of participants into the study); (ii) at intervention (bias in classification of interventions); and (iii) post-intervention (bias due to deviations from intended interventions; bias due to missing data; bias in measurement of outcomes; bias in selection of the reported results). Two of the authors (JA and HS) independently assessed the risk of bias. Any disagreement in the rating was resolved through discussion and by a third author (FMC).

### Summary Measures, Synthesis of Results, and Publication Bias

We followed previously established methods (Clemente et al., 2021). Briefly, analysis and interpretation of results were only conducted in the case of at least three studies provided baseline and follow-up data for the same measure. Pre-training and post-training means and standard deviations (SD) for dependent variables were used to calculate effect sizes (ES; Hedge's *g*) for each outcome measure in the SSGs and control groups. Data were standardized using post-intervention SD values. The random-effects model was used to account for differences between studies that might impact the SSG-based effect (Deeks et al., 2008; Kontopantelis et al., 2013). The ES values are presented with 95% confidence intervals (CI). Calculated ES were interpreted using the following scale: <0.2, trivial; 0.2–0.6, small; >0.6–1.2, moderate; >1.2–2.0, large; >2.0–4.0, very large; >4.0, extremely large (Hopkins et al., 2009). Heterogeneity was assessed using the *I*^2^ statistic, with values of <25%, 25–75%, and >75% considered to represent low, moderate, and high levels of heterogeneity, respectively (Higgins and Thompson, 2002). The risk of bias was explored using the extended Egger's test (Egger et al., 1997). When bias was present, the trim and fill method was applied (Duval and Tweedie, 2000), in which case L0 was assumed as the default estimator for missing studies (Shi and Lin, 2019). All analyses were carried out using the Comprehensive Meta-Analysis software (version 2; Biostat, Englewood, NJ, USA). Statistical significance was set at *p* ≤ 0.05.

Moderated analyses were planned to use a random-effects model and independently calculated single factor analysis. When possible, the median split technique was planned (Moran et al., 2019b). Moderator analysis was considered for the total session during interventions.

## Results

### Study Identification and Selection

**Figure 1:**
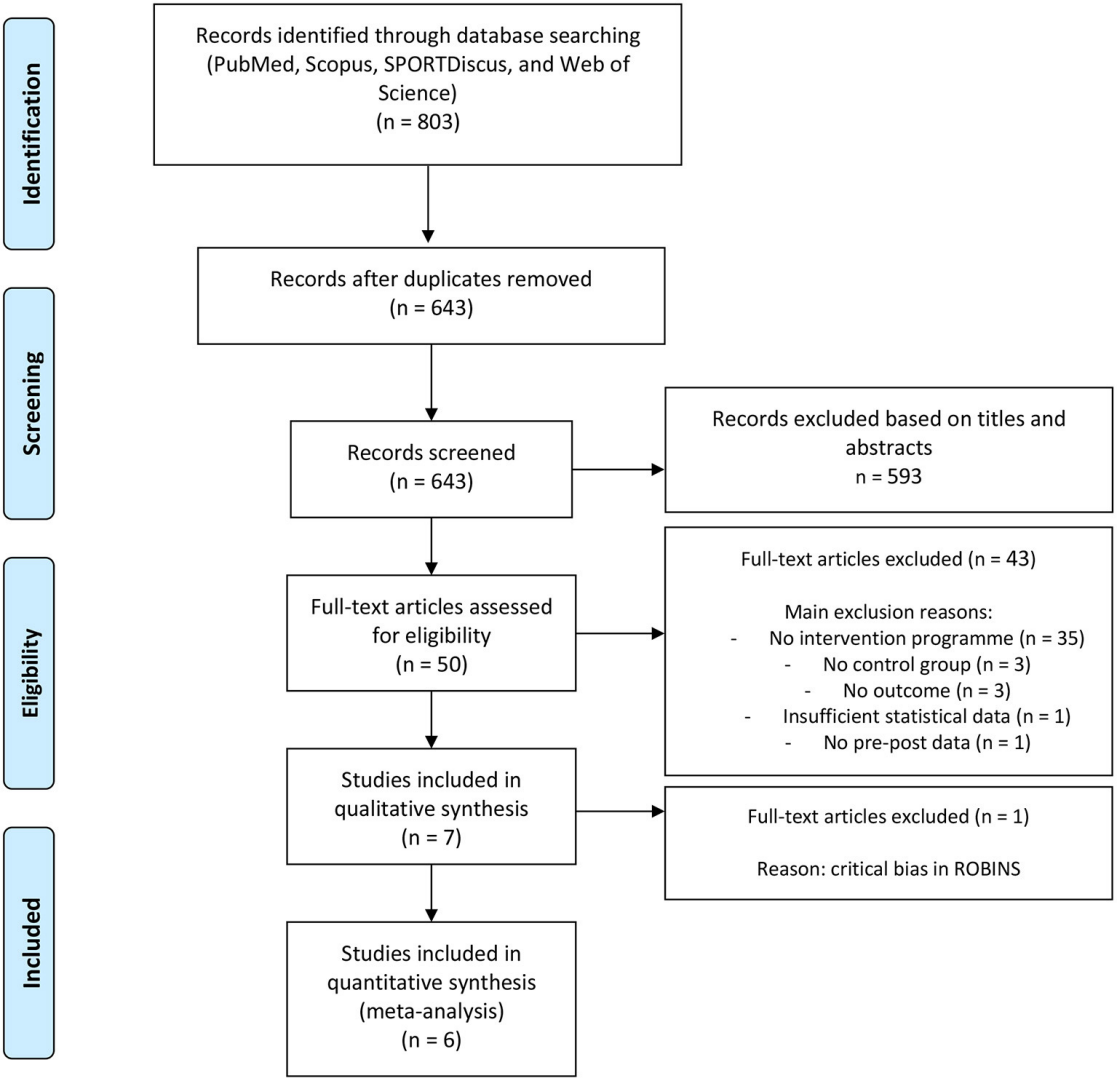
PRISMA flow diagram highlighting the selection process for the studies included in the current systematic review.

The searching of databases identified an initial 803 titles. Duplicates (160 references) were subsequently removed either automatically or manually. The remaining 643 articles were screened for their relevance based on titles and abstracts, resulting in the removal of a further 593 studies. The full texts of the remaining 50 articles were examined diligently. After reading full texts, a further 43 studies were excluded owing to a number of reasons: did not develop an intervention program (n=35); lack of control group (*n* = 3); did not included technical/tactical outcome (*n* = 3); insufficient statistical data (*n* = 1); did not reported pre-post data (*n* = 1). After methodological assessment, one article was excluded by critical risk of bias. None of the included articles were written in other language than English. Six articles were eligible for the systematic review and meta-analysis (Figure 1). The included articles provided mean and standard deviation pre-post-training data for at least the main outcome.

### Methodological Quality

The randomized-controlled trials included in this study were analyzed with the RoB2 tool and the results can be found in Table 2.

**Table 2:** Assessment of risk of bias of randomized trials with The Risk of Bias 2 (RoB2).

	**D1**	**D2**	**D3**	**D4**	**D5**	**Overall**	**Direction (overall)**
Chaouachi et al., 2014	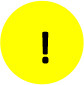	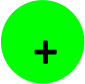	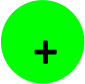	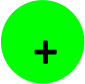	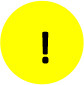	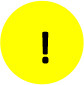	NA
Radziminski et al., 2013	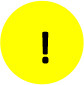	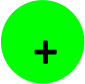	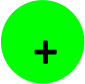	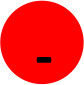	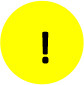	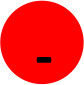	Favors experimental
Trajkovic et al., 2017	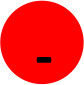	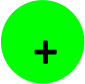	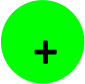	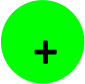	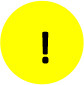	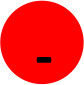	Favors experimental

*D1: randomization process; D2: Deviations from the intended interventions; D3: Missing outcome data; D4: Measurement of the outcome; D5: Selection of the reported result. Green: low risk; Yellow: Some concerns; Red: high risk; NA: not applicable; Direction provide information about the tendency of methodological favoring*.

The assessment of the non-randomized studies intervention studies can be found in Table 3. Since one article (Práxedes et al., 2016) presented overall critical evaluation (based on the assessment criteria of the instrument), and in accordance with Cochrane manual (Higgins et al., 2019), was not included in qualitative and quantitative synthesis.

**Table 3:** Assessment of risk of bias of non-randomized trials with ROBINS-I.

**Study**	**D1**	**D2**	**D3**	**D4**	**D5**	**D6**	**D7**	**Overall**
Arslan et al., 2020	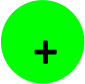	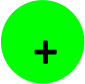	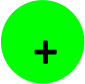	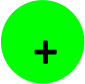	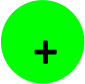	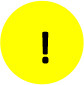	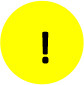	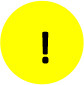
Daga et al., 2020	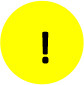	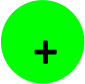	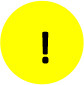	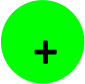	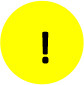	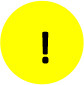	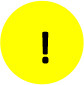	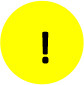
Jastrzebski et al., 2014	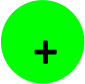	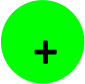	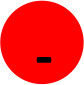	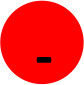	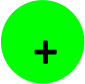	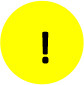	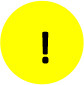	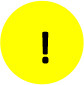
Práxedes et al., 2016	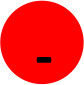	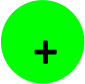	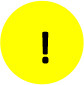	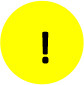	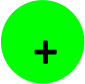	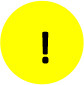	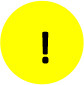	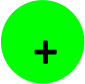

*D1: reaching risk of bias judgements for bias due to confounding; D2: reaching risk of bias judgments in selection of participants into the study; D3: reaching risk of bias judgments for bias in classification of interventions; D4: reaching risk of bias judgments for bias due to deviations from intended interventions; D5: reaching risk of bias judgements for bias due to missing data; D6: reaching risk of bias judgements for bias in measurement of outcomes; D7: reaching risk of bias judgments for bias in selection of the reported result; Green: low risk; Yellow: moderate/serious risk; Red: critical risk*.

### Study Characteristics

Although only three of the six included articles in the qualitative and quantitative synthesis were randomized (Radziminski et al., 2013; Chaouachi et al., 2014; Daga et al., 2020), none of the six included articles reported significant differences between control ad experimental groups at baseline. The characteristics of the six studies included in the meta-analysis can be found in Table 4. Additionally, the details of the SSGs-based programs can be found in Table 5 and the other training interventions in Table 6. The three included randomized-controlled studies involved three individual experimental groups and 43 participants, and 55 participants in the four control groups. The three included non-randomized studies involved three individual experimental groups and 39 participants, and 35 participants in the three control groups.

**Table 4:** Characteristics of the included studies and outcomes extracted.

**Study**	**N**	**Mean age (yo)**	**Experience (y)**	**Sex**	**Team Sport**	**Randomized**	**Design**	**Control**	**Sig. Dif. Baseline**	**Variables assessed in the study and tendency**	**Tests or tools used**	**Outcome extracted**
Arslan et al., 2020	SSG: 11 ActCon: 10	14.2	3.3–3.5	Male	Soccer	No	Parallel	Running-based group	No	Agility with ball (s) – less is better	Zig-zag agility with ball	TE: agility with ball
Chaouachi et al., 2014	SSG: 12 ActCon: 12 PassCon: 12	14.2	ND	Male	Soccer	Yes	Parallel	ActCon: multidirectional sprints PassCon: only field training	No	Reactive agility test with ball (s)–less is better	Reactive agility test with ball	TE: reactive agility test with ball
Daga et al., 2020	SSG: 17 ActCon: 14	9.0	ND	Male	Soccer	No	Parallel	Multilateral training	No	Shuttle dribble test–less is better	Shuttle dribble test	TE: shuttle dribble test
Jastrzebski et al., 2014	SSG: 11 ActCon: 11	15.8	6–7	Male	Soccer	No	Parallel	Running-based group	No	DFB test (n) –high is better	Technical skill battery of the German Soccer Federation	TE: DFB
Radziminski et al., 2013	SSG: 9 ActCon: 11	15.1	5–7	Male	Soccer	Yes	Parallel	Running-based group	No	DFB test (n) – high is better	Technical skill battery of the German Soccer Federation	TE: DFB
Trajkovic et al., 2017	SSG: 22 ActCon: 20	11.2	2.1–2.2	Female	Volleyball	Yes	Parallel	Instructional training	No	Overhead pass Forearm pass Setting Serving Serving under fatigue —high is better	Observational test	TE: overhead pass

*N, number of participants in the study; Yo, years old; Y, years; M, men; W, women; h, hour; ActCon, active control group; PassCon, passive control group; Sig. Dif. Baseline, significant differences at baseline; ND, not-described; CT, controlled trial; RCT, randomized controlled trial; TE, technical execution; TB, tactical behavior; DFB, German Soccer Federation test*.

**Table 5:** Characteristics of the SSG-intervention programs in the included studies.

**Study**	**Duration (w)**	**d/w**	**Total sessions**	**SSG formats**	**SSG pitch dimension (length × width)**	**SSG area per player (m^**2**^)**	**Other conditions**	**Sets**	**Reps**	**Work duration**	**Work intensity**	**Between reps duration**	**Type of recovery**
Arslan et al., 2020	5	2	10	2 vs. 2	20 × 15-m	75 m^2^	ND	2	2	2.5–4.5 min	ND	2 min	Passive
Daga et al., 2020	12	2	24	2 vs. 1, 2 vs.2, 3 vs.3, 4 vs.2	ND	ND	ND	ND	ND	ND	ND	ND	ND
Chaouachi et al., 2014	6	3	18	1 vs.1, 2vs.2 and 3vs.3	10 × 20, 20 × 20 and 20 × 30-m	100 m^2^	Ball contacts restricted (2-3)	1–2	2–4	30 s−2 min	80–85% HRmax	2 min	ND
Jastrzebski et al., 2014	8	2	16	3 vs.3	18 × 30-m	90 m^2^	ND	ND	7	3 min	NR	90 s	Active recovery
Radziminski et al., 2013	8	2	16	3 vs.3 or 3 vs.3 +1	18 × 30-m	77–90 m^2^	ND	ND	5	4 min	>90% HRmax	3 min	Light activity
Trajkovic et al., 2017	12	3	36	2 vs.2 and 3 vs.3	7 × 3 and 12 × 6-m	5 m^2^ and 12 m^2^	ND	ND	ND	ND	ND	ND	ND

*d/w, days per week; SSG, small-sided games; TGfU, questioning used as strategy for promotion of tactical learning; ND, not described; HRmax, maximal Heart Rate*.

**Table 6:** Characteristics of the other training programs in the included studies.

**Study**	**Characteristics**
Arslan et al., 2020	Running-based HIIT was implemented twice a week over 5 weeks. Two sets of 6–10 min of 15”−15” running at 90–95% VIFT were performed.
Daga et al., 2020	The multilateral approach consisted of 25 min per session in which the players developed physical qualities using multilateral or general exercises such as sprint, relays, jumps over the hurdle. The ball was not included.
Chaouachi et al., 2014	The change-of-direction group performed preplanned COD drills (e.g., skipping, 5-0-5 meters, half-*T*-test 20 m, shuttle 4 × 10 m. The ball was not included.
Jastrzebski et al., 2014	The interval running group performed seven 3 min (15”−15”) runs with 90 s of active recovery. The ball was not included.
Radziminski et al., 2013	The interval running protocol consisted in 5 intervals of 4 min of running, interspaced by 3 min of active recovery.
Trajkovic et al., 2017	The instructional training sessions have used blocked practice, performing individual skills against the wall or to a partner in non-competitive environment, multiple repetitions, and practice of technique in a closed-skill context.

*HIIT, high-intensity interval training; VIFT, final velocity at 30–15 intermittent fitness test*.

### SSGs vs. Control: Effects on Technical Execution

A summary of the included studies and results of technical execution reported before and after SSGs-based compared to control intervention are provided in Table 7.

**Table 7:** Summary of the included studies and results of technical execution before and after intervention.

**Study**	**Group**	***N***	**Before Mean ± SD**	**After Mean ± SD**	After-before (%)^*^	**Tendency of change**
Arslan et al., 2020	SSG	11	8.85 ± 0.54	8.36 ± 0.53	−5.5	Beneficial
Chaouachi et al., 2014	SSG	12	2.65 ± 0.09	2.45 ± 0.11	−7.5	Beneficial
Daga et al., 2020	SSG	17	15.76 ± 1.13	12.75 ± 1.52	−19.1	Beneficial
Jastrzebski et al., 2014	SSG	11	325.2 ± 43.9	342.5 ± 33.0	5.3	Beneficial
Radziminski et al., 2013	SSG	9	307.8 ± 59.0	341.0 ± 50.2	10.8	Beneficial
Trajkovic et al., 2017	SSG	22	5.24 ± 1.56	6.73 ± 1.79	28.4	Beneficial
Arslan et al., 2020	CON	10	8.56 ± 0.34	8.45 ± 0.36	−1.3	Beneficial
Chaouachi et al., 2014	CON^a^	12	2.67 ± 0.18	2.54 ± 0.16	−4.9	Beneficial
Daga et al., 2020	CON	14	14.00 ± 1.29	12.07 ± 0.97	−13.8	Beneficial
Jastrzebski et al., 2014	CON	11	333.4 ± 34.11	344.0 ± 34.6	3.2	Beneficial
Radziminski et al., 2013	CON	11	302.3 ± 40.4	319.6 ± 35.7	5.7	Beneficial
Chaouachi et al., 2014	CON^b^	12	2.68 ± 0.08	2.55 ± 0.08	−4.9	Beneficial
Trajkovic et al., 2017	CON	20	6.36 ± 2.29	6.59 ± 1.92	3.6	Beneficial

a, active control; b, passive control. SSG, small-sided games group; CON, control group^*^, although some values reflects negative and other positive values (depending on the outcome measure), all changes denotes favorable changes in both the SSG and CON groups; SD, standard-deviation.

**Figure 2:**
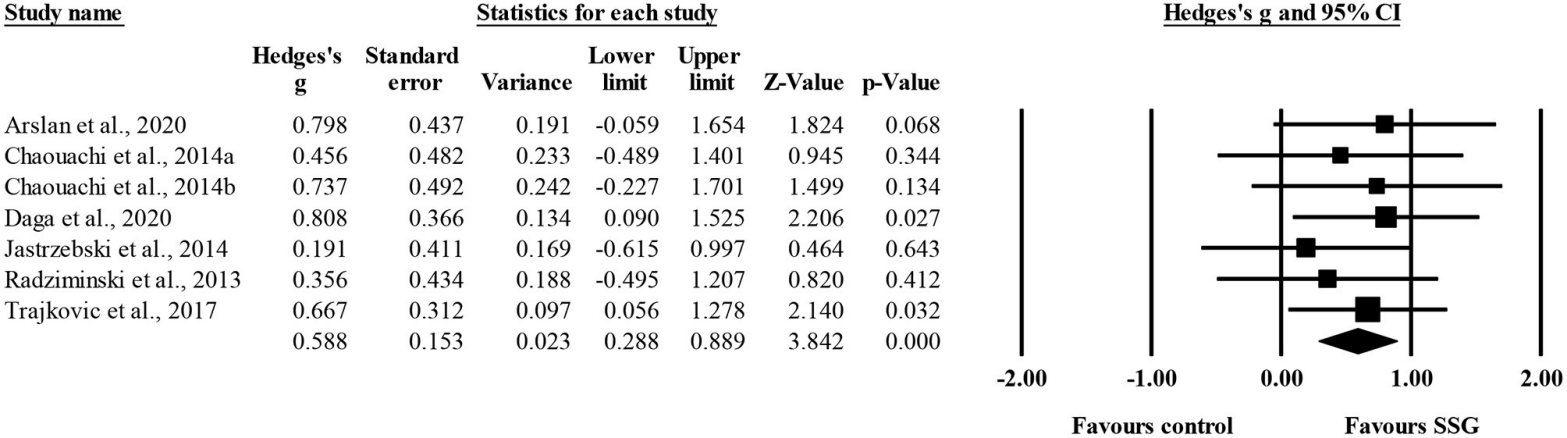
Forest plot of changes in technical execution, in youth athletes from team sports participating in SSG-based programs (intervention) compared to controls. Values shown are effect sizes (Hedges's g) with 95% confidence intervals (CI). The size of the plotted squares reflects the statistical weight of the study. The black diamond reflects the overall result.

Six studies provided data for technical execution, involving six experimental and seven control groups (pooled *n* = 172). Results showed a small effect of SSGs on technical execution (ES = 0.59; 95% CI = 0.29 to 0.89; *p* < 0.001; *I*^2^ = 0.0%; Egger's test *p* = 0.590; Figure 2) when compared to controls.

The single training factor analysis revealed a similar (*p* = 0.433) moderate (ES = 0.68, four study groups) and small (ES = 0.44, three study groups) improvement in technical execution after >17 and <17 SSG training sessions, respectively.

**Figure 3:**
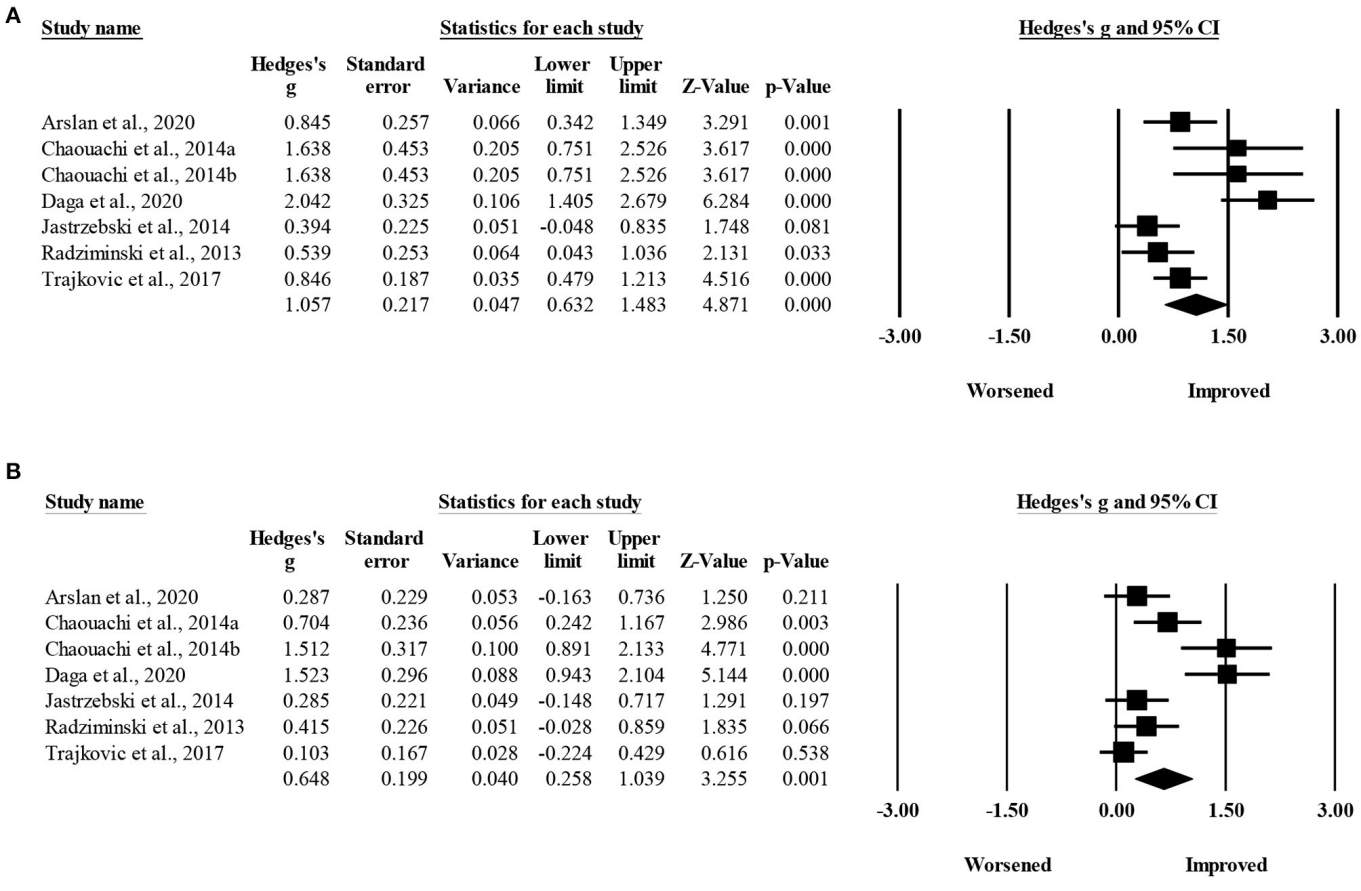
Forest plot of within-group pre-post intervention changes in technical execution, in youth athletes from team sports participating in **(A)** SSGs-based programs and **(B)** control condition. Values shown are effect sizes (Hedges's g) with 95% confidence intervals (CI). The size of the plotted squares reflects the statistical weight of the study. The black diamond reflects the overall result.

The within-group meta-analysis showed a moderate effect on technical execution after both SSGs (six groups, pooled *n* = 82; ES = 0.106; 95% CI = 0.63 to 1.48; *p* < 0.001; *I*^2^ = 75.9%; Egger's test *p* = 0.090; Figure 3A), and control conditions (seven groups, pooled *n* = 90; ES = 0.65; 95% CI = 0.26 to 1.04; *p* = 0.001; *I*^2^ = 80.3%; Egger's test *p* = 0.003, unchanged after the trim and fill method was applied; Figure 3B).

### SSGs vs. Control: Effects on Tactical Behavior

None of the six included studies presented tactical behavior-related outcomes. In screening only one study presented tactical behavior outcome (Práxedes et al., 2016), although was excluded based on a critical score obtained after using the ROBINS tool.

## Discussion

This study aimed to assess the effects of SSG programs on the technical execution and tactical behaviors of youth team sports players. The results indicated positive and small effects of training programs with SSGs on technical execution. No studies regarding tactical outcomes were included in the review. The discussion will be divided into topics, each regarding one of the two outcomes selected for this review (technical execution and tactical behaviors).

### SSGs vs. Control: Effects on Technical Execution

SSGs were effective for improving players' technical execution in the selected studies. This result is in line with the assumption that SSGs might nurture youth sports players' technical development, as they provide players with a game-based environment in which they are constantly required to apply motor actions to solve emerging problems. Interestingly, all the selected studies (Radziminski et al., 2013; Chaouachi et al., 2014; Jastrzebski et al., 2014; Trajkovic et al., 2017; Arslan et al., 2020; Daga et al., 2020) adopted formats ranging from 1 vs. 1 to 3 vs. 3. These smaller SSG formats were reported to increase players' ball engagement and provide more variable stimuli (Clemente et al., 2019; Caso and van der Kamp, 2020), which might explain the performance enhancements. The current review, therefore, reinforces the positive role that SSGs play in technical development and encourages coaches to adopt this tool in their training programs.

Although positive adaptations in technical execution were reported when comparing control vs. intervention groups, the magnitude of these differences was small. We argue that the type of measurement regarding technical execution might explain this reduced statistical effect. Specifically, most of the adopted tests are based on measuring agility with the ball or change-of-direction abilities (Radziminski et al., 2013; Chaouachi et al., 2014; Jastrzebski et al., 2014; Arslan et al., 2020; Daga et al., 2020), while others adopted a battery of closed-environment tests (Radziminski et al., 2013; Jastrzebski et al., 2014). In both cases, the measurements were taken out of the game context. Contrarily, in SSGs, technical executions are always integrated with tactical decisions, so larger differences between control and intervention groups could be expected if the measurements were taken in a more representative game-based scenario. Interestingly, a previous study found no significant correlations between in-game tactical-technical dribbling performance and players' dribbling performance in a technical test without the game context (Praça et al., 2015), reinforcing the previous rationale.

Also, the performance in the agility test is influenced by a cognitive aspect (perceptual and decision-making processes) and by players' abilities to quickly change direction (Young et al., 2015; Pojskic et al., 2018). Therefore, technical execution is not directly measured. Consequently, players might experience an improvement in the testing performance, which is not fully explained by an improvement in ball dribbling technical execution. Therefore, the magnitude of the difference between control and experimental groups is expected to be reduced. For both mentioned reasons, future studies should look to develop and adopt valid and reliable in-game-based instruments to measure improvements in technical execution.

Moderate pre-to-post improvements were observed within control and intervention groups. While improving technical execution is an expected outcome for the intervention group, the same did not apply to the control groups. Besides the abovementioned issues regarding the tests and measurements, we argue that this unexpected outcome is linked to the way the training programs were designed. Specifically, most of the selected studies included game-based activities (in which technical executions were required) in the training program of the control group as players took part in sport-specific team training (Radziminski et al., 2013; Chaouachi et al., 2014; Jastrzebski et al., 2014; Trajkovic et al., 2017; Arslan et al., 2020). In the selected studies, however, there was no information regarding the content of such sport-specific team training.

Considering how training programs are usually developed, we might expect players to engage in technical execution training, which explains the improvement in the control group. However, as the magnitude of the difference was higher in the intervention groups than in the control ones (ES 1.06 and 0.65, respectively), the SSGs boosted the technical execution enhancements attained through regular training regimens, which reinforces their positive role. However, as high heterogeneity between the studies was observed in the control and intervention groups, generalizations are not recommended and future studies are required.

Finally, a similar small-to-moderate improvement in technical execution was observed after >17 and <17 SSG training sessions. Although longer interventions are expected to generate greater improvements, two issues might explain the current results. While one study (Trajkovic et al., 2017) included 36 sessions, all the other >17 training session interventions varied from 18 to 24 sessions. Therefore, the magnitude of the difference in the length of the training program was likely to be not enough to elicit different adaptations. Moreover, none of the selected studies introduced non-arbitrary pedagogical strategies to control and progress the training contents in the intervention group. Adjusting the task complexity to players' current levels is expected to facilitate the learning (Machado et al., 2020). Therefore, the absence of a control condition for the training process might have reduced the expected impact of the longest intervention programs on players' technical execution.

While the abovementioned rationale seems to explain the current results, the two longest intervention programs (Trajkovic et al., 2017; Daga et al., 2020) were coincidently the two most statistically weighted studies in the meta-analysis (see Figure 2), as they showed the most relevant intervention impact on the responses (see hedge's G and 95% CI) and the lowest variance across the studies.

In team sports, learning is not a linear process, and differences are age and maturity-dependent (Fransen et al., 2017). For this reason, although the current analysis did not show differences between >17 and <17 SSG training session programs, short training programs might not be enough to favor the development of technical skills in late-developed players, who require longer training periods to achieve such improvements.

### SSGs vs. Control: Effects on Tactical Behavior

No selected study examined the effects of SSGs on tactical behavior. This result indicates the need for further research on this topic for an evidence-based prescription. We acknowledge that the absence of evidence must not be understood as evidence of absence, as previous game-based approaches that are strongly based on small-sided games have been effective in nurturing team sports players' tactical skills (Kinnerk et al., 2018). There are two main possible causes for this absence of studies: the difficulty in choosing instruments to evaluate tactical behaviors and the challenge of designing interventions with high external validity in team sports.

Previous instruments used to evaluate tactical behaviors are largely based on observational designs (Oslin et al., 1998; Costa et al., 2011; González-Víllora et al., 2015). Although this low-cost strategy allows performance indicators to be obtaining in team sports, there are issues regarding their reliability, as they account for discrete events. Specifically, low-frequency events are less likely to present a good test-retest reliability, which increases the variability of the responses and reduces the power of the analysis. For example, weak intersession intraclass correlation coefficients were reported for the frequency of tactical principles in a previous study in soccer (Bredt et al., 2016).

Moving toward contemporary technologies, such as positional analysis, might allow the researchers to access more reliable measures, which will encourage the development of intervention studies. Previous studies adopted this perspective using linear and nonlinear modeling to detect players' and teams' behaviors in game-based contexts (Ramos et al., 2017; Ribeiro et al., 2020) and showed interesting pathways for future research.

### Limitations, Future Research and Practical Applications

This review provides interesting practical applications for coaches and researchers. Specifically, because significant and beneficial effects of using SSG training interventions for improving technical execution were observed, coaches are encouraged to systematically include SSGs in training programs to nurture players' technical skills. During SSGs, players experience high variability in their actions, which may also provide players with a more adaptative technical pattern in line with the demands of the matches in team sports.

On the other hand, limitations must be accounted for. From the selected studies, five addressed improvements in technical execution in soccer, while only one included volleyball athletes. Therefore, although our conclusions seem to be more generalizable to soccer, studies in other sports are required. Also, the studies did not include players of different age groups and did not include elite youth players. As the technical development of high-level athletes might be different from novice youth-academy players, future studies in elite groups are recommended. Also, in-game technical execution assessments are still required, as there is no evidence that the SSGs are more effective than control training for improving in-game performance. Finally, constantly developing valid and reliable instruments to assess players' behaviors related to game-based tasks will benefit researchers and practitioners by increasing the quality of the information used to adjust training contents and complexity.

The only selected study that considered tactical behaviors was excluded due to methodological issues. The lack of control for confounding variables might have biased the results, which reduced the methodological quality and demands caution when interpreting such results. As there were no selected studies regarding players' tactical behavior development after SSGs-based training programs, we encourage researchers to search for suitable experimental designs to address this issue.

Finally, integrated analysis covering the impacts of SSG-based on programs in multiple dimensions (e.g., physiological/physical, technical, and tactical) must be considered, namely integrating into the analysis the dose-response effects by monitoring the load imposed (Gomez-Carmona et al., 2018; Gómez-Carmona et al., 2019) and the accumulated effects of technical/tactical responses to specific drills (Fernández-Espínola et al., 2020).

## Conclusions

This systematic review revealed the significant and beneficial effects of using SSG training interventions to improve technical execution in comparison to control groups. Interestingly, the sub-group analysis revealed that using more or less than 17 training sessions had similar effects on the improvement of technical execution. The within-group analysis had great heterogeneity; thus, any generalization of beneficial effects should be avoided. Interestingly, a search for the effects of SSG training interventions on tactical behaviors yielded no eligible articles. Therefore, future research should consider tactical behaviors as outcomes to be assessed during SSG interventions.

## Data Availability Statement

The raw data supporting the conclusions of this article will be made available by the authors, without undue reservation.

## Author Contributions

FC lead the project, wrote and revised the original manuscript. RR-C analyzed and interpreted the data, wrote the statistical report, and revised the original manuscript. FC and HS run the data search, performed the methodological assessment, conducted the data extraction, wrote, and revised the original manuscript. GP, JA, AS, TR, and BK wrote and revised the original manuscript. All authors contributed to the article and approved the submitted version.

## Conflict of Interest

The authors declare that the research was conducted in the absence of any commercial or financial relationships that could be construed as a potential conflict of interest.

## References

[B1] Arslan E. Orer G. Clemente F. (2020). Running-based high-intensity interval training vs. small-sided game training programs: effects on the physical performance, psychophysiological responses and technical skills in young soccer players. Biol. Sport 37, 165–173. 10.5114/biolsport.2020.94237 32508384 PMC7249797

[B2] Bredt S. G. T. Praça G. M. Figueiredo L. S. de Paula L. V. Silva P. C. R. de Andrade A. G. P.. (2016). Reliability of physical, physiological and tactical measures in small-sided soccer games with numerical equality and numerical superiority. Rev. Bras. Cineantropometria & Desempenho Hum. 18:602–10. 10.5007/1980-0037.2016v18n5p602

[B3] Bujalance-Moreno P. Latorre-Roman P. A. Garcia-Pinillos F. (2019). A systematic review on small-sided games in football players: acute and chronic adaptations. J. Sports Sci. 37, 921–949. 10.1080/02640414.2018.1535821 30373471

[B4] Caso S. van der Kamp J. (2020). Variability and creativity in small-sided conditioned games among elite soccer players. Psychol. Sport Exerc. 48:101645. 10.1016/j.psychsport.2019.101645

[B5] Chaouachi A. Chtara M. Hammami R. Chtara H. Turki O. Castagna C. (2014). Multidirectional sprints and small-sided games training effect on agility and change of direction abilities in Youth Soccer. J. Strength Cond. Res. 28, 3121–3127. 10.1519/JSC.0000000000000505 25148467

[B6] Clemente F. (2019). The threats of small-sided soccer games: a discussion about their differences with the match external load demands and their variability levels. Strength Cond. J., 1:526. 10.1519/SSC.0000000000000526

[B7] Clemente F. M. (2016). Small-sided and conditioned games in basketball training: a review filipe manuel clemente. Strength Cond. J. 38, 49–58. 10.1519/SSC.0000000000000225

[B8] Clemente F. M. Afonso J. Castillo D. Arcos A. L. Silva A. F. Sarmento H. (2020). The effects of small-sided soccer games on tactical behavior and collective dynamics: a systematic review. Chaos Solitons Fractals 134:109710. 10.1016/j.chaos.2020.109710

[B9] Clemente F. M. Chen Y. S. Bezerra J. P. Guiomar J. Lima R. (2018). Between-format differences and variability of technical actions during small-sided soccer games played by young players. Hum. Mov. 19, 114–120. 10.5114/hm.2018.83103

[B10] Clemente F. M. Ramirez-Campillo R. Nakamura F. Y. Sarmento H. (2021). Effects of high-intensity interval training in men soccer player's physical fitness: A systematic review with meta-analysis of randomized-controlled and non-controlled trials. J. Sports Sci. Ahead-of-p, 1–22. 10.1080/02640414.2020.1863644 33423603

[B11] Clemente F. M. Sarmento H. (2020). The effects of small-sided soccer games on technical actions and skills: a systematic review. Hum. Mov. 21, 100–119. 10.5114/hm.2020.93014

[B12] Clemente F. M. Sarmento H. Costa I. T. Enes A. R. Lima R. (2019). Variability of technical actions during small-sided games in young soccer players. J. Hum. Kinet. 69, 201–212. 10.2478/hukin-2019-0013 31666902 PMC6815080

[B13] Collaboration C. (2016). Data extraction template for included studies. Available online at: https://cccrg.cochrane.org/sites/cccrg.cochrane.org/files/public/uploads/det_2015_revised_final_june_20_2016_nov_29_revised.doc (accessed January 2, 2021).

[B14] Costa I. T. Garganta J. Greco P. J. Mesquita I. Maia J. (2011). System of tactical assessment in Soccer (FUT-SAT): development and preliminary validation. Motricidade 7, 69–84. 10.6063/motricidade.7(1).121

[B15] Daga F. A. Baseggio L. Gollin M. Beratto L. (2020). Game-based versus multilateral approach: effects of a 12-week program on motor skill acquisition and physical fitness development in soccer school children. J. Sports Med. Phys. Fitness 60:10726. 10.23736/S0022-4707.20.10726-6 32432448

[B16] Davids K. Araújo D. Correia V. Vilar L. (2013). How small-sided and conditioned games enhance acquisition of movement and decision-making skills. Exerc. Sport Sci. Rev. 41, 154–161. 10.1097/JES.0b013e318292f3ec 23558693

[B17] Deeks J. J. Higgins J. P. Altman D. G. (2008). Analysing data and undertaking meta-analyses, in Cochrane Handbook for Systematic Reviews of Interventions, eds Higgins J. P. Green S. (The Cochrane Collaboration), 243–296. 10.1002/9780470712184.ch9

[B18] Duval S. Tweedie R. (2000). Trim and fill: a simple funnel-plot-based method of testing and adjusting for publication bias in meta-analysis. Biometrics 56, 455–463. 10.1111/j.0006-341X.2000.00455.x 10877304

[B19] Egger M. Smith G. D. Schneider M. Minder C. (1997). Bias in meta-analysis detected by a simple, graphical test. BMJ 315, 629–634. 10.1136/bmj.315.7109.629 9310563 PMC2127453

[B20] Fernández-Espínola C. Robles M. T. A. Fuentes-Guerra F. J. G. (2020). Small-sided games as a methodological resource for team sports teaching: a systematic review. Int. J. Environ. Res. Public Health 17. 10.3390/ijerph17061884 32183219 PMC7143077

[B21] Fransen J. Bennett K. J. M. Woods C. T. French-Collier N. Deprez D. Vaeyens R.. (2017). Modelling age-related changes in motor competence and physical fitness in high-level youth soccer players: implications for talent identification and development. Sci. Med. Footb. 1, 203–208. 10.1080/24733938.2017.1366039

[B22] Gomez-Carmona C. D. Gamonales J. M. Pino-Ortega J. Ibanez S. J. (2018). Comparative analysis of load profile between small-sided games and official matches in youth soccer players. Sport. (Basel, Switzerland) 6:6040173. 10.3390/sports6040173 30545050 PMC6316437

[B23] Gómez-Carmona C. D. Gamonales-Puerto J. M. Feu S. Ibáñez S. J. (2019). Estudio de la carga interna y externa a través de diferentes instrumentos. Un estudio de casos en fútbol formativo. Sport. Sci. J. Sch. Sport. Phys. Educ. Psychomot. 5, 444–468. 10.17979/sportis.2019.5.3.5464

[B24] González-Víllora S. Serra-Olivares J. Pastor-Vicedo J. C. da Costa I. T. (2015). Review of the tactical evaluation tools for youth players, assessing the tactics in team sports: football. Springerplus 4:663. 10.1186/s40064-015-1462-0 26558166 PMC4630321

[B25] Green S. Higgins J. (2005). Cochrane Handbook for Systematic Reviews of Interventions. Hoboken, NJ: John Wiley and Sons.

[B26] Hammami A. Gabbett T. J. Slimani M. Bouhlel E. (2018). Does small-sided games training improve physical-fitness and specific skills for team sports? A systematic review with meta-analysis. J. Sports Med. Phys. Fitness 58, 1446–1455. 10.23736/S0022-4707.17.07420-5 29072025

[B27] Higgins J. P. Thomas J. Chandler J. Cumpston M. Li T. Page M. J.. (2019). Cochrane Handbook for Systematic Reviews of Interventions, 2nd Edn. Chichester: John Wiley and Sons. 10.1002/9781119536604

[B28] Higgins J. P. T. Thompson S. G. (2002). Quantifying heterogeneity in a meta-analysis. Stat. Med. 21, 1539–1558. 10.1002/sim.1186 12111919

[B29] Hopkins W. G. Marshall S. W. Batterham A. M. Hanin J. (2009). Progressive statistics for studies in sports medicine and exercise science. Med. Sci. Sport. Exerc. 41, 3–13. 10.1249/MSS.0b013e31818cb278 19092709

[B30] Jastrzebski Z. Barnat W. Dargiewicz R. Jaskulska E. Szwarc A. Radzimiński Ł. (2014). Effect of In-season generic and soccer-specific high-intensity interval training in young soccer players. Int. J. Sports Sci. Coach. 9, 1169–1179. 10.1260/1747-9541.9.5.1169

[B31] Kinnerk P. Harvey S. MacDonncha C. Lyons M. (2018). A review of the game-based approaches to coaching literature in competitive team sport settings. Quest 70, 401–418. 10.1080/00336297.2018.1439390

[B32] Kontopantelis E. Springate D. A. Reeves D. (2013). A re-analysis of the cochrane library data: the dangers of unobserved heterogeneity in meta-analyses. PLoS ONE 8:e69930. 10.1371/journal.pone.0069930 23922860 PMC3724681

[B33] Machado J. C. Barreira D. Teoldo I. Serra-Olivares J. Góes A. José Scaglia A. (2020). Tactical behaviour of youth soccer players: differences depending on task constraint modification, age and skill level. J. Hum. Kinet. 75, 225–238. 10.2478/hukin-2020-0051 33312309 PMC7706672

[B34] Moher D. Liberati A. Tetzlaff J. Altman D. G. (2009). Preferred reporting items for systematic reviews and meta-analyses: the PRISMA Statement. PLoS Med. 6:e1000097. 10.1371/journal.pmed.1000097 19621072 PMC2707599

[B35] Moran J. Blagrove R. C. Drury B. Fernandes J. F. T. Paxton K. Chaabene H.. (2019a). Effects of small-sided games vs. conventional endurance training on endurance performance in male youth soccer players: a meta-analytical comparison. Sport. Med. 49, 731–742. 10.1007/s40279-019-01086-w 30868441

[B36] Moran J. Clark C. C. T. Ramirez-Campillo R. Davies M. J. Drury B. (2019b). A meta-analysis of plyometric training in female youth. J. Strength Cond. Res. 33, 1996–2008. 10.1519/JSC.0000000000002768 30052601

[B37] Olthof S. B. H. Frencken W. G. P. Lemmink K. A. P. M. (2018). Match-derived relative pitch area changes the physical and team tactical performance of elite soccer players in small-sided soccer games. J. Sports Sci. 36, 1557–1563. 10.1080/02640414.2017.1403412 29125029

[B38] Ometto L. Vasconcellos F. V. Cunha F. A. Teoldo I. Souza C. R. B. Dutra M. B.. (2018). How manipulating task constraints in small-sided and conditioned games shapes emergence of individual and collective tactical behaviours in football: a systematic review. Int. J. Sports Sci. Coach. 13, 1200–1214. 10.1177/1747954118769183

[B39] Oslin J. L. Mitchell S. A. Griffin L. L. (1998). The game performance assesment instrument (GPAI): development and preliminary validation. J. Teach. Phys. Educ. 17, 231–243. 10.1123/jtpe.17.2.231

[B40] Pojskic H. Åslin E. Krolo A. Jukic I. Uljevic O. Spasic M.. (2018). Importance of reactive agility and change of direction speed in differentiating performance levels in junior soccer players: reliability and validity of newly developed soccer-specific tests. Front. Physiol. 9:506. 10.3389/fphys.2018.00506 29867552 PMC5962722

[B41] Praça G. M. M. Soares V. V. O. V. Matias C. J. A. S. Costa I. T. Greco P. J. J. da Costa I. T.. (2015). Relationship between tactical and technical performance in youth soccer players. Rev. Bras. Cineantropometria e Desempenho Hum. 17, 136–144. 10.5007/1980-0037.2015v17n2p136

[B42] Práxedes A. Del Villar F. Pizarro D. Moreno A. (2018a). the impact of nonlinear pedagogy on decision-making and execution in youth soccer players according to game actions. J. Hum. Kinet. 62, 185–198. 10.1515/hukin-2017-0169 29922390 PMC6006529

[B43] Práxedes A. Moreno A. Gil-Arias A. Claver F. Del Villar F. (2018b). The effect of small-sided games with different levels of opposition on the tactical behaviour of young footballers with different levels of sport expertise. PLoS ONE 13:e0190157. 10.1371/journal.pone.0190157 29320519 PMC5761879

[B44] Práxedes A. Moreno A. Sevil J. García-González L. Del Villar F. (2016). A Preliminary study of the effects of a comprehensive teaching program, based on questioning, to improve tactical actions in young footballers. Percept. Mot. Skills 122, 742–756. 10.1177/0031512516649716 27207601

[B45] Radziminski L. Rompa P. Barnat W. Dargiewicz R. Jastrzebski Z. (2013). A comparison of the physiological and technical effects of high-intensity running and small-sided games in young soccer players. Int. J. Sports Sci. Coach. 8, 455–465. 10.1260/1747-9541.8.3.455

[B46] Ramos J. Lopes R. J. Marques P. Araújo D. (2017). Hypernetworks reveal compound variables that capture cooperative and competitive interactions in a soccer match. Front. Psychol. 8:01379. 10.3389/fpsyg.2017.01379 28894427 PMC5581353

[B47] Ribeiro J. Lopes R. Silva P. Araújo D. Barreira D. Davids K.. (2020). A multilevel hypernetworks approach to capture meso-level synchronisation processes in football. J. Sports Sci. 38, 494–502. 10.1080/02640414.2019.1707399 31876443

[B48] Sarmento H. Clemente F. M. Harper L. D. Teoldo I. Owen A. Figueiredo A. J.. (2018). Small sided games in soccer—a systematic review. Int. J. Perform. Anal. Sport 00, 1–57. 10.1080/24748668.2018.1517288

[B49] Serra-Olivares J. Clemente F. M. González-Víllora S. (2016). Tactical expertise assessment in youth football using representative tasks. Springerplus 5:2955. 10.1186/s40064-016-2955-1 27547675 PMC4978649

[B50] Shi L. Lin L. (2019). The trim-and-fill method for publication bias. Med. (Baltimore). 98:e15987. 10.1097/MD.0000000000015987 31169736 PMC6571372

[B51] Silva A. F. Conte D. Clemente F. M. (2020). Decision-making in youth team-sports players: a systematic review. Int. J. Environ. Res. Public Health 17:3803. 10.37766/inplasy2020.4.0207 32471126 PMC7312689

[B52] Sterne J. A. Hernán M. A. Reeves B. C. Savović J. Berkman N. D. Viswanathan M.. (2016). ROBINS-I: a tool for assessing risk of bias in non-randomised studies of interventions. BMJ 355:i4919. 10.1136/bmj.i4919 27733354 PMC5062054

[B53] Sterne J. A. C. Savović J. Page M. J. Elbers R. G. Blencowe N. S. Boutron I.. (2019). RoB 2: a revised tool for assessing risk of bias in randomised trials. BMJ 366:l4898. 10.1136/bmj.l4898 31462531

[B54] Trajkovic N. Kristicevic T. Sporis G. (2017). Small-sided games vs. instructional training for improving skill accuracy in young female volleyball players. Acta Kinesiol. 11, 72–76. Available online at: http://actakinesiologica.com/04cl13nt/

[B55] Turner A. Walker S. Stembridge M. Coneyworth P. Reed G. Birdsey L.. (2011). A testing battery for the assessment of fitness in soccer players. Strength Cond. J. 33, 29–39. 10.1519/SSC.0b013e31822fc80a33166289

[B56] Young W. Dawson B. Henry G. J. (2015). Agility and change-of-direction speed are independent skills: implications for training for agility in invasion sports. Int. J. Sports Sci. Coach. 10, 159–169. 10.1260/1747-9541.10.1.159

